# Supplementary material for: Who falls between the cracks? Identifying eligible PrEP users among people with Sub-Saharan African migration background living in Antwerp, Belgium
Source: PLoS One. 2021 Aug 18;16(8):e0256269. doi: 10.1371/journal.pone.0256269 (PMC8372948; doi:10.1371/journal.pone.0256269)
Supplement: S1 Table — (DOCX) [file pone.0256269.s001.docx]

**S1 Table. Percentages of SAM meeting the eligibility criteria (weighted data)**

|  | | | | | | |
| --- | --- | --- | --- | --- | --- | --- |
|  |  | **Total (N = 685)** | | | | |
|  | **Eligibility criteria** | N | N total  (excl. missings) | Valid  % | | % of total N  (incl. missings) |
| 1. | Had sex with at least four different partners the last 12 months | 75 | 663 | 11.3 | | 11.0 |
|  | and the last time was condomless | **45** | **618** | **7.2** | | **6.5** |
| 2. | Had a STI less than 6 months ago | **11** | **685** | **1.7** | | **1.7** |
| 4. | Used alcohol during last sexual activity | 110 | 663 | 16.6 | | 16.0 |
|  | and this time was condomless | 65 | 658 | 9.9 | | 9.5 |
|  | Used drugs during last sexual activity | 14 | 686 | 2.1 | | 2.1 |
|  | and this time was condomless | 12 | 686 | 1.7 | | 1.7 |
|  | Used drugs and/or alcohol during last sexual activity | 118 | 663 | 17.8 | | 17.2 |
|  | and this time was condomless | **72** | **658** | **10.9** | | **10.5** |
| 6. | Had transactional sex in the last 12 months | 44 | 636 | | 6.9 | 6.4 |
|  | and the last time was condomless | **20** | **636** | | **3.1** | **2.9** |
| 7.1 | Concurrent relationship | 124 | 602 | | 20.5 | 18.1 |
|  | and the last time was condomless | 70 | 602 | | 11.6 | 10.2 |
|  | and not planning to use a condom in the future (low condom use intentions) | **15** | **602** | | **2.6** | **2.3** |
| 7.2 | The origin of the last sex partner was African | 459 | 634 | | 72.5 | 67.0 |
|  | and no knowledge about his/her HIV status | 165 | 615 | | 26.8 | 24.1 |
|  | and the last time sex with this partner was condomless | **101** | **615** | | **16.4** | **14.7** |
| 7.3 | Sexually active during traveling to Africa (less than 12 months ago) | 73 | 682 | | 10.7 | 10.7 |
|  | with a casual partner (traveling with or meeting there) or a stable partner living in Africa | 55 | 669 | | 8.2 | 8.0 |
|  | and it was condomless | 26 | 664 | | 4.0 | 3.9 |
|  | Sexually active during traveling to Europe (less than 12 months ago) | 41 | 684 | | 6.0 | 6.0 |
|  | with a casual partner or a stable partner living in that visiting country | 36 | 681 | | 5.3 | 5.3 |
|  | and it was condomless | 12 | 681 | | 1.7 | 1.7 |
|  | Sexually active during travelling to Africa/Europe (< 12 months ago) with a casual  partner or a stable partner living in Africa/that visiting country and it was condomless | **37** | **662** | | **5.6** | **5.4** |
| TOTAL | Meeting at least one of the criteria (= eligible to use PrEP) | 201 | 663 | | 30.3 | 29.6 |
|  | one criterion | 132 | 663 | | 19.9 | 19.2 |
|  | two criteria | 47 | 663 | | 7.1 | 6.8 |
|  | three criteria | 15 | 663 | | 2.2 | 2.1 |
| Meeting at least one of the RIZIV criteria (excluding the SAM specific criteria: 7.1, 7.2, 7.3) | | 117 | 663 | | 17.7 | 17.1 |
