# Supplementary material for: Who falls between the cracks? Identifying eligible PrEP users among people with Sub-Saharan African migration background living in Antwerp, Belgium
Source: PLoS One. 2021 Aug 18;16(8):e0256269. doi: 10.1371/journal.pone.0256269 (PMC8372948; doi:10.1371/journal.pone.0256269)
Supplement: S2 Table — (DOCX) [file pone.0256269.s002.docx]

**S2 Table. Eligibility criteria associated to sociodemographic, economic, migration related factors and forced sex**

|  | **>= 4 sex partners** | | | | | | | | **STI last 6 months** | | | | | | | | **Psychoactive substances during sex** | | | | | | | |
| --- | --- | --- | --- | --- | --- | --- | --- | --- | --- | --- | --- | --- | --- | --- | --- | --- | --- | --- | --- | --- | --- | --- | --- | --- |
|  | Unadjusted | | |  | Adjusted | | |  | Unadjusted | | |  | Adjusted | | |  | Unadjusted | | |  | Adjusted | | |  |
| Variable | OR | 95%-CI | | | OR | 95%-CI | | | OR | 95%-CI | | | OR | 95%-CI | | | OR | 95%-CI | | | OR | 95%-CI | | |
| **Age** |  |  |  |  |  |  |  |  |  |  |  |  |  |  |  |  |  |  |  |  |  |  |  |  |
| Between 18-30 years old (ref.) | | | | |  |  |  |  |  |  |  |  |  |  |  |  |  |  |  |  |  |  |  |  |
| Between 31-40 years old | 1.4 | 0.7 | 2.7 |  | 1.4 | 0.7 | 3.0 |  | 1.9 | 0.3 | 11.4 |  | 3.3 | 0.5 | 22.8 |  | 1.9 | 1.1 | 3.4 | * | 2.4 | 1.3 | 4.7 | ** |
| Older than 41 years old | 0.2 | 0.1 | 0.8 | * | 0.2 | 0.1 | 0.8 | * | 5.1 | 1.0 | 26.4 |  | 13.0 | 2.0 | 82.9 | ** | 1.0 | 0.5 | 2.1 |  | 1.5 | 0.7 | 3.2 |  |
| **Gender** |  |  |  |  |  |  |  |  |  |  |  |  |  |  |  |  |  |  |  |  |  |  |  |  |
| Women (ref.) | |  |  |  |  |  |  |  |  |  |  |  |  |  |  |  |  |  |  |  |  |  |  |  |
| Men | 4.5 | 1.8 | 11.5 | ** | 5.3 | 2.0 | 14.0 | *** | 0.9 | 0.3 | 3.2 |  | 1.0 | 0.3 | 3.9 |  | 1.3 | 0.8 | 2.2 |  | 1.4 | 0.7 | 2.5 |  |
| **MSM** |  |  |  |  |  |  |  |  |  |  |  |  |  |  |  |  |  |  |  |  |  |  |  |  |
| No MSM (ref.) | |  |  |  |  |  |  |  |  |  |  |  |  |  |  |  |  |  |  |  |  |  |  |  |
| MSM | 1.4 | 0.2 | 9.1 |  | 0.7 | 0.1 | 5.2 |  | ^a^ |  |  |  |  |  |  |  |  |  |  |  |  |  |  |  |
| **Relation status** | |  |  |  |  |  |  |  |  |  |  |  |  |  |  |  |  |  |  |  |  |  |  |  |
| Not in a relationship (ref.) | | | |  |  |  |  |  |  |  |  |  |  |  |  |  |  |  |  |  |  |  |  |  |
| In a relation and cohabiting | 0.8 | 0.4 | 1.5 |  | 1.3 | 0.6 | 2.7 |  | 0.2 | 0.0 | 1.3 |  | 0.1 | 0.0 | 0.9 | * | 0.5 | 0.3 | 0.9 | * | 0.6 | 0.3 | 1.1 |  |
| In a relation and not cohabiting | 0.9 | 0.4 | 2.1 |  | 1.2 | 0.5 | 3.1 |  | 1.2 | 0.3 | 4.6 |  | 1.4 | 0.3 | 5.9 |  | 0.6 | 0.3 | 1.3 |  | 0.7 | 0.4 | 1.6 |  |
| **Region of origin** | | | |  |  |  |  |  |  |  |  |  |  |  |  |  |  |  |  |  |  |  |  |  |
| Western Africa (ref.) | | |  |  |  |  |  |  |  |  |  |  |  |  |  |  |  |  |  |  |  |  |  |  |
| Central Africa | 1.0 | 0.5 | 2.0 |  | 1.2 | 0.6 | 2.7 |  | 0.2 | 0.0 | 2.3 |  | 0.2 | 0.0 | 2.7 |  | 2.1 | 1.2 | 3.6 | ** | 3.5 | 1.9 | 6.5 | *** |
| Southern or Eastern Africa | 0.5 | 0.1 | 2.7 |  | 0.3 | 0.1 | 1.9 |  | 2.6 | 0.5 | 12.6 |  | 2.7 | 0.4 | 16.9 |  | 5.8 | 2.7 | 12.5 | *** | 6.3 | 2.7 | 14.6 | *** |
| **Legal status** | |  |  |  |  |  |  |  |  |  |  |  |  |  |  |  |  |  |  |  |  |  |  |  |
| Documented (ref.) | | |  |  |  |  |  |  |  |  |  |  |  |  |  |  |  |  |  |  |  |  |  |  |
| Probably undocumented | 3.9 | 2.1 | 7.3 | *** | 3.7 | 1.8 | 7.5 | *** | 1.9 | 0.6 | 6.7 |  | 1.6 | 0.4 | 6.8 |  | 1.8 | 1.1 | 3.1 | ** | 1.4 | 0.8 | 2.7 |  |
| **Education** |  |  |  |  |  |  |  |  |  |  |  |  |  |  |  |  |  |  |  |  |  |  |  |  |
| Primary school or less (ref.) | | | | |  |  |  |  |  |  |  |  |  |  |  |  |  |  |  |  |  |  |  |  |
| Completed secondary | 1.5 | 0.6 | 4.0 |  | 1.1 | 0.4 | 3.1 |  | 0.4 | 0.1 | 1.3 |  | 0.4 | 0.1 | 1.6 |  | 1.8 | 0.8 | 4.1 |  | 1.7 | 0.7 | 4.0 |  |
| Continued education | 1.3 | 0.5 | 3.5 |  | 0.9 | 0.3 | 2.8 |  | 0.1 | 0.0 | 0.8 | * | 0.1 | 0.0 | 1.1 |  | 1.5 | 0.7 | 3.6 |  | 1.0 | 0.4 | 2.5 |  |
| **Financial problems** | | |  |  |  |  |  |  |  |  |  |  |  |  |  |  |  |  |  |  |  |  |  |  |
| No (ref.) |  |  |  |  |  |  |  |  |  |  |  |  |  |  |  |  |  |  |  |  |  |  |  |  |
| Sometimes/most of the time | 1.2 | 0.6 | 2.3 |  | 0.9 | 0.4 | 1.8 |  | 0.7 | 0.2 | 2.3 |  | 0.4 | 0.1 | 1.7 |  | 1.9 | 1.1 | 3.3 | * | 2.0 | 1.1 | 3.8 | * |
| **Forced sex (lifetime)** | | |  |  |  |  |  |  |  |  |  |  |  |  |  |  |  |  |  |  |  |  |  |  |
| Never (ref.) |  |  |  |  |  |  |  |  |  |  |  |  |  |  |  |  |  |  |  |  |  |  |  |  |
| Ever | 1.0 | 0.3 | 3.3 |  | 1.7 | 0.5 | 6.1 |  |  |  |  |  |  |  |  |  | 1.5 | 0.7 | 3.7 |  | 2.0 | 0.8 | 5.1 |  |

| (continued) | | | | | | | | | | | | | | | | |
| --- | --- | --- | --- | --- | --- | --- | --- | --- | --- | --- | --- | --- | --- | --- | --- | --- |
|  | **Transactional sex** | | | | | | | | **Concurrent relation + low condom use intensions** | | | | | | | |
|  | Unadjusted | | |  | Adjusted | | |  | Unadjusted | | |  | Adjusted | | |  |
|  | OR | 95%-CI | | | OR | 95%-CI | | | OR | 95%-CI | | | OR | 95%-CI | | |
| **Age** |  |  |  |  |  |  |  |  |  |  |  |  |  |  |  |  |
| Between 18-30 years old (ref.) |  |  |  |  |  |  |  |  |  |  |  |  |  |  |  |  |
| Between 31-40 years old | 1.6 | 0.6 | 4.4 |  | 1.3 | 0.4 | 4.2 |  | 0.7 | 0.2 | 2.4 |  | 1.2 | 0.3 | 4.2 |  |
| Older than 41 | 0.9 | 0.2 | 3.1 |  | 1.8 | 0.4 | 7.8 |  | 0.9 | 0.2 | 3.0 |  | 2.4 | 0.6 | 10.1 |  |
| **Gender** |  |  |  |  |  |  |  |  |  |  |  |  |  |  |  |  |
| Women (ref.) |  |  |  |  |  |  |  |  |  |  |  |  |  |  |  |  |
| Men | 1.3 | 0.4 | 3.4 |  | 1.0 | 0.3 | 2.9 |  | 0.4 | 0.2 | 1.2 |  | 0.4 | 0.1 | 1.3 |  |
| **MSM** |  |  |  |  |  |  |  |  |  |  |  |  |  |  |  |  |
| No MSM (ref.) |  |  |  |  |  |  |  |  |  |  |  |  |  |  |  |  |
| MSM | 24.1 | 7.0 | 83.5 | *** | 23.8 | 4.8 | 119.3 | *** | 9.0 | 1.2 | 65.9 | * | 33.7 | 2.5 | 452.2 | ** |
| **Relation status** |  |  |  |  |  |  |  |  |  |  |  |  |  |  |  |  |
| Not in a relationship (ref.) |  |  |  |  |  |  |  |  |  |  |  |  |  |  |  |  |
| In a relation and cohabiting | 0.2 | 0.0 | 0.8 | * | 0.2 | 0.0 | 1.0 |  | 0.1 | 0.0 | 0.6 | * | 0.1 | 0.0 | 0.6 | * |
| In a relation and not cohabiting | 1.7 | 0.6 | 4.4 |  | 2.0 | 0.7 | 6.0 |  | 0.5 | 0.1 | 2.0 |  | 0.5 | 0.1 | 2.0 |  |
| **Region of origin** |  |  |  |  |  |  |  |  |  |  |  |  |  |  |  |  |
| Western Africa (ref.) |  |  |  |  |  |  |  |  |  |  |  |  |  |  |  |  |
| Central Africa | 0.9 | 0.3 | 2.7 |  | 1.9 | 0.5 | 6.4 |  | 0.2 | 0.0 | 1.6 |  | 0.2 | 0.0 | 1.9 |  |
| Southern or Eastern Africa | 2.5 | 0.6 | 10.3 |  | 2.1 | 0.4 | 11.1 |  | 3.8 | 1.1 | 13.5 | * | 4.2 | 1.0 | 18.3 |  |
| **Legal status** |  |  |  |  |  |  |  |  |  |  |  |  |  |  |  |  |
| Documented (ref.) |  |  |  |  |  |  |  |  |  |  |  |  |  |  |  |  |
| Probably undocumented | 3.9 | 1.6 | 9.6 | ** | 2.8 | 0.9 | 8.7 |  | 2.5 | 0.9 | 7.1 |  | 1.8 | 0.6 | 6.2 |  |
| **Education** |  |  |  |  |  |  |  |  |  |  |  |  |  |  |  |  |
| Primary school or less (ref.) |  |  |  |  |  |  |  |  |  |  |  |  |  |  |  |  |
| Completed secondary | 0.7 | 0.2 | 2.5 |  | 0.9 | 0.2 | 3.7 |  | 0.4 | 0.1 | 1.1 |  | 0.4 | 0.1 | 1.5 |  |
| Continued education | 1.2 | 0.3 | 4. |  | 1.4 | 0.3 | 6.0 |  | 0.2 | 0.1 | 0.9 |  | 0.4 | 0.1 | 1.9 |  |
| **Financial problems** |  |  |  |  |  |  |  |  |  |  |  |  |  |  |  |  |
| No (ref.) |  |  |  |  |  |  |  |  |  |  |  |  |  |  |  |  |
| Sometimes/most of the time | 1.6 | 0.6 | 4.3 |  | 1.4 | 0.4 | 4.5 |  | 0.9 | 0.3 | 2.4 |  | 0.7 | 0.2 | 2.1 |  |
| **Forced sex (lifetime)** |  |  |  |  |  |  |  |  |  |  |  |  |  |  |  |  |
| Never (ref.) |  |  |  |  |  |  |  |  |  |  |  |  |  |  |  |  |
| Ever | 2.9 | 0.9 | 10.0 |  | 2.7 | 0.6 | 11.4 |  | 2.3 | 0.5 | 10.3 |  | 0.8 | 0.1 | 5.6 |  |

| (continued) | | | | | | | | | | | | | | | | |
| --- | --- | --- | --- | --- | --- | --- | --- | --- | --- | --- | --- | --- | --- | --- | --- | --- |
|  | **African sex partner + unknown HIV-status** | | | | | | | | **Sexual active during travelling** | | | | | | | |
|  | Unadjusted | | |  | Adjusted | | |  | Unadjusted | | |  | Adjusted | | |  |
|  | OR | 95%-CI | | | OR | 95%-CI | | | OR | 95%-CI | | | OR | 95%-CI | | |
| **Age** |  |  |  |  |  |  |  |  |  |  |  |  |  |  |  |  |
| Between 18-30 years old (ref.) |  |  |  |  |  |  |  |  |  |  |  |  |  |  |  |  |
| Between 31-40 years old | 1.0 | 0.6 | 1.7 |  | 1.3 | 0.7 | 2.3 |  | 2.1 | 0.8 | 5.0 |  | 3.3 | 1.0 | 10.1 | * |
| Older than 41 | 1.3 | 0.8 | 2.3 |  | 1.9 | 1.0 | 3.6 | * | 3.0 | 1.1 | 7.4 | * | 4.9 | 1.5 | 15.5 | ** |
| **Gender** |  |  |  |  |  |  |  |  |  |  |  |  |  |  |  |  |
| Women (ref.) |  |  |  |  |  |  |  |  |  |  |  |  |  |  |  |  |
| Men | 1.1 | 0.7 | 1.7 |  | 1.5 | 0.9 | 2.5 |  | 4.4 | 1.6 | 11.7 | ** | 3.6 | 1.3 | 10.3 | * |
| **MSM** |  |  |  |  |  |  |  |  |  |  |  |  |  |  |  |  |
| No MSM (ref.) |  |  |  |  |  |  |  |  |  |  |  |  |  |  |  |  |
| MSM | 2.4 | 0.7 | 7.9 |  | 1.6 | 0.4 | 6.5 |  | 2.7 | 0.5 | 13.8 |  | 1.2 | 0.2 | 7.3 |  |
| **Relation status** |  |  |  |  |  |  |  |  |  |  |  |  |  |  |  |  |
| Not in a relationship (ref.) |  |  |  |  |  |  |  |  |  |  |  |  |  |  |  |  |
| In a relation and cohabiting | 0.6 | 0.4 | 1.0 | * | 0.5 | 0.3 | 0.9 | * | 1.2 | 0.6 | 2.7 |  | 1.1 | 0.4 | 2.8 |  |
| In a relation and not cohabiting | 0.7 | 0.4 | 1.3 |  | 0.6 | 0.3 | 1.2 |  | 2.5 | 1.1 | 5.8 | * | 3.3 | 1.3 | 8.8 | * |
| **Region of Origin** |  |  |  |  |  |  |  |  |  |  |  |  |  |  |  |  |
| Western Africa (ref.) |  |  |  |  |  |  |  |  |  |  |  |  |  |  |  |  |
| Central Africa | 0.8 | 0.5 | 1.3 |  | 1.2 | 0.7 | 2.1 |  | 1.1 | 0.5 | 2.3 |  | 0.8 | 0.3 | 2.0 |  |
| Southern or Eastern Africa | 1.8 | 0.8 | 3.8 |  | 1.9 | 0.8 | 4.3 |  | 0.9 | 0.2 | 3.9 |  | 0.9 | 0.2 | 4.6 |  |
| **Legal status** |  |  |  |  |  |  |  |  |  |  |  |  |  |  |  |  |
| Documented (ref.) |  |  |  |  |  |  |  |  |  |  |  |  |  |  |  |  |
| Probably undocumented | 1.2 | 0.7 | 1.9 |  | 1.0 | 0.6 | 1.8 |  | 1.1 | 0.5 | 2.4 |  | 1.1 | 0.4 | 2.8 |  |
| **Education** |  |  |  |  |  |  |  |  |  |  |  |  |  |  |  |  |
| Primary school or less (ref.) |  |  |  |  |  |  |  |  |  |  |  |  |  |  |  |  |
| Completed secondary | 0.6 | 0.3 | 1.0 |  | 0.6 | 0.4 | 1.2 |  | 0.8 | 0.3 | 2.1 |  | 0.8 | 0.3 | 2.2 |  |
| Continued education | 0.3 | 0.2 | 0.6 | *** | 0.3 | 0.2 | 0.6 | *** | 1.2 | 0.5 | 3.2 |  | 0.8 | 0.3 | 2.3 |  |
| **Financial problems** |  |  |  |  |  |  |  |  |  |  |  |  |  |  |  |  |
| No (ref.) |  |  |  |  |  |  |  |  |  |  |  |  |  |  |  |  |
| Sometimes/most of the time | 1.3 | 0.8 | 2.0 |  | 1.2 | 0.7 | 1.9 |  | 1.2 | 0.6 | 2.6 |  | 1.2 | 0.5 | 2.6 |  |
| **Forced sex (lifetime)** |  |  |  |  |  |  |  |  |  |  |  |  |  |  |  |  |
| Never (ref.) |  |  |  |  |  |  |  |  |  |  |  |  |  |  |  |  |
| Ever | 2.3 | 1.2 | 4.8 | * | 2.4 | 1.1 | 5.3 | * | 0.9 | 0.2 | 3.6 |  | 1.1 | 0.2 | 5.0 |  |
| *p<0.05 **p<0.01 ***p<0.001  ^a^ No valuable results available because the number of cases were too low (gray colored) | | | | | | | | | | | | | | | | |
