# Supplementary material for: Who falls between the cracks? Identifying eligible PrEP users among people with Sub-Saharan African migration background living in Antwerp, Belgium
Source: PLoS One. 2021 Aug 18;16(8):e0256269. doi: 10.1371/journal.pone.0256269 (PMC8372948; doi:10.1371/journal.pone.0256269)
Supplement: S1 File — (DOCX) [file pone.0256269.s003.docx]

**S1 File. Interpretation of the results of the logistic regression analyses for the separate eligibility criteria**

The same logistic regression models were estimated for each eligibility criterion separately, but excluding the variables *unstable housing*, *employment status*, *migration duration*, and *study setting* as these were not significant for any of the criteria. The results also did not change during the stepwise procedure and thus only the third model is presented in S2 Table in addition to the unadjusted ORs.

The results show that SSA migrants without health insurance were more likely to have four or more sex partners and to report condomless sex. Without controlling for the other variables, they were also more likely to report condomless transactional sex. Male SSA migrants reporting same sex behavior (MSM) also had a higher likelihood of transactional condomless sex and of being in a concurrent relationship with condomless sex and low likelihood of condom use in the future, also after taking the other variables into account.

SSA migrants of the age-group between 31 to 40 years, from Central, Southern or Eastern Africa, or those experiencing economic hardship, were more likely to take drugs and/or alcohol while having condomless sex compared to younger SSA migrants, SSA migrants from Western Africa and SSA migrants without economic hardship. Taking the other variables into account, SSA migrants older than 40 were more likely to have had a STI during the last six months, to have reported condomless sex during travelling and condomless sex with a partner of African origin without knowing his/her HIV status, but they were less likely to have had four or more sexual partners the last 12 months compared to the youngest age group (below 30 years of age).
