# Supplementary material for: Who falls between the cracks? Identifying eligible PrEP users among people with Sub-Saharan African migration background living in Antwerp, Belgium
Source: PLoS One. 2021 Aug 18;16(8):e0256269. doi: 10.1371/journal.pone.0256269 (PMC8372948; doi:10.1371/journal.pone.0256269)
Supplement: Attachment — Submitted filename: Plosone_Response_to_reviewers_13July2021.docx [file pone.0256269.s006.docx]

**Rebuttal letter**

13^th^ of July 2021

Dear Editor-in-Chief,

***“Who falls between the cracks? Identifying eligible PrEP users among people with Sub-Saharan African migration background living in Antwerp, Belgium”***

Thank you for the opportunity to revise this article for publication in *PLOS ONE*. The amended manuscript reflects the authors’ efforts to address the additional minor comments and suggestions raised by the reviewer.

The remainder of this letter gives specific details of how we addressed each comment, together with the line numbers (in the manuscript with track changes) upon which the relevant changes appear.

As you indicated in your communication, the first reviewer is satisfied with our previous adaptations and the second reviewer has identified a few minor areas that need additional clarification and editing. We also respond to the remaining editorial comments.

Yours sincerely,

Dr. Veerle Buffel

**Reviewer #1:**

Thank you for the clarification. I have no additional comments. The publication can be published. Best Wishes

**Reviewer #2:**

Thank you for giving me the opportunity to review this revised manuscript. I thought it was well written and the subject is interesting. I do have some questions and point of feedback that will hopefully help to further improve manuscript.

*Major:*
**Comment 1:** Line 163-165 & results 272-287: While I do understand why this extra analysis is interesting, I found that it was a distraction from the main question the authors wanted to answer in this paper and it made the Results section a bit confusing and long. I would suggest to either take these analyses out of the paper or move the results to the Supplements to keep the paper concise.

*We agree with the remark of the reviewer, as this was also the reason why the results of these extra analyses were already only displayed as supplementary material (S2 Table. Eligibility criteria associated to sociodemographic, economic, migration related factors and forced sex). According to the suggestion of the reviewer, we have replaced also the interpretation of these results to the Supplements (S3 Supporting information. Interpretation of the results of the logistic regression analyses for the separate eligibility criteria). (Lines 231-234)*

*We employed logistic regression models using a stepwise approach: to socio-demographic and migration related factors (Model 1) we added socio-economic factors (Model 2) and in the last step we also included the variable forced sex (Model 3). To get a better understanding of each HIV risk factor, the same analyses were also done with the* *individual eligibility criteria as dependent dichotomous variables. These results and their interpretation can be found respectively in S2 Table and S3 Supporting information.*

**Comment 2:** Line 196-198: I did not fully understand what the authors meant by control variable study setting. Do you mean this variable was controlled for in multivariable analysis? Did you expect differences between different study setting? A suggestion would be to use multilevel analysis to account for this.

*In general, we did not expect differences in ‘eligibility to PrEP use’ across the study settings, as the respondents were randomly selected among the people present at the study setting at time of recruitment. The study settings were chosen during formative research as places were people originating from SSA often get together. Only for some specific risk factors, we could expect some differences: for example, people recruited at a bar or party could have a higher likelihood to meet the risk factor ‘using psychoactive substances during condomless sex’ than people recruited at the church (this was also confirmed in the bivariate analyses).*

*Indeed, we also considered a multilevel analysis, but there was no significant variance at the higher level (study setting) and there were no higher level variables we want to estimate on ‘PrEP eligibility’. Also the results of the bivariate analyses (Chi^2^-tests) showed no significant differences between the study settings in terms of ‘eligibility to PrEP use’. In addition, by controlling for study setting through the use of dummies, we were able to assess the differences between the respondents in terms of ‘eligibility to use PrEP’ according to the study setting where they were recruited. Because of these reasons we prefer a one-level logistic regression analysis with the inclusion of the variable ‘study setting’ over a two-level analysis with study setting as the higher level to account for the clustering of and potential similarities between participants recruited from the same study setting.*

*In the revised manuscript we have added some information about the two-stage time location sampling and the selection of study settings (sites where SSA migrants often get together). This has made it easier to explain clearly the control variable ‘study setting’. (lines 106-110 and 206-210)*

*The TOGETHER data are the most recent behavioral data available for this sub-population in Belgium. The study used a two-stage time-location sampling (TLS) to obtain a venue-based sample of n=744 adult sub-Saharan African migrants in Antwerp (1). A TLS takes advantage of the fact that some hard-to-reach populations tend to gather at certain types of sites/clusters at certain times. A list of these sites was prepared in a formative study and formed the sampling frame: at the first level, clusters (or sites) were randomly selected with a probability proportional-to-size and at the second level, a fixed number of study participants were randomly selected from each cluster.*

*…*

*The study sites where study participants were selected were categorized in five types of settings and this is included as control variable: bars/parties of African organization, churches, public place (park, street, square), events and meetings of African organizations, and other (e.g. shop, hair salon, library, asylum center)(1).*

*Minor:*
**Comment 3:** Line 50-52: Could you add some more context here on the number of new HIV infections in 2019 and what proportion of new HIV infections in 2019 was among heterosexual individuals?

Based on a recent report of Sciensano (the public health institution of Belgium), we have added some more information about the new HIV infections in 2019 and the proportion of heterosexual transmissions among these new HIV cases in Belgium. (Lines 50-55)

***Introduction***

*People with a Sub-Saharan Africa (SSA) migration background are the second largest group affected by HIV in Belgium (1).* *In 2019, a total of 923 new HIV diagnoses were identified in Belgium, among them 349 were through heterosexual transmission (51% of the cases with known transmission type) (2). People with SSA migration background constituted 48% of all new HIV cases with a heterosexual transmission mode in 2019, 67% among them were women (2). …*

*2. Sasse A, Deblonde J, De Rouck M, Montourcy M, Van Beckhoven D. Epidemiologie van aids en hiv-infectie in België toestand op 31 december 2019 [The epidemiology of AIDS and HIV infection in Belgian: the situation at the 31th of December 2019], Brussels: Sciensano; 2020.*

**Comment 4:** Line 66: Is it a box or a Figure? It now says Fig 1. Box 1.

This figure is a box, but according to the submission system of the Journal we could only include this box as ‘figure’

**Comment 5:** Line 101: what do you mean by two-stage time location sampling?

A 2-stage time location sampling (TLS) takes advantage of the fact that some hard-to-reach populations tend to congregate at certain types of locations. A list of these locations (settings) is prepared in a formative study and forms the sampling frame from which a 2-stage cluster probability sample was selected. At the first level of sampling clusters (or sites) were randomly selected with a probability proportional-to-size and at the second level, from each cluster a random selection of a fixed number of study participants was done. In the revised manuscript we have briefly explained the TLS and for more detailed information we refer to the protocol paper of the Together project. (lines 106-110)

*The TOGETHER data are the most recent behavioral data available for this sub-population in Belgium. The study used a two-stage time-location sampling (TLS) to obtain a venue-based sample of n=744 adult Sub-Saharan African migrants in Antwerp (1).* *A TLS takes advantage of the fact that some hard-to-reach populations tend to gather at certain types of sites/clusters at certain times. A list of these sites was prepared in a formative study and formed the sampling frame: at the first level, clusters (or sites) were randomly selected with a probability proportional-to-size and at the second level, a fixed number of study participants were randomly selected from each cluster.*

*All individuals socializing in a given setting at the time of the study visit (available attendance data) were eligible for inclusion in the survey if they met the following criteria: (1) self-identified sub-Saharan African migrant; (2) age 18 years or above; (3) accepting to answer the questionnaire; (4) accepting to provide an oral fluid sample; and (5) providing written informed consent. Recruitment, data collection and weighting procedures to adjust for unequal selection probability are described elsewhere (1, 16).*

**Comment 6:** Line 212-214: Were all variables assessed in univariable analysis included in the multivariable model? Were all variables kept in the model when going to the next Model (i.e. were all variables from Model 1, still in Model 2)?

*Yes, we have used a stepwise procedure. This means that we have added variables to the next models, while the other variables of the previous model remained included.*

**Comment 7:** Line 229: do you mean multivariable regression analysis instead of multiple?

*Yes, indeed, thanks for remarking this. It is changed in the revised manuscript.*

**Comment 8:** Line 250: please use multivariable here unless you added multiple outcome variables to your model.

*We have changed it to ‘multivariable’ because the analysis has multiple independent variables but only one outcome variable.*

**Journal Requirements:**

Please review your reference list to ensure that it is complete and correct. If you have cited papers that have been retracted, please include the rationale for doing so in the manuscript text, or remove these references and replace them with relevant current references. Any changes to the reference list should be mentioned in the rebuttal letter that accompanies your revised manuscript. If you need to cite a retracted article, indicate the article’s retracted status in the References list and also include a citation and full reference for the retraction notice.

*We have revised the reference list according to the PLOSONE guidelines.*
